# Endometrial Surveillance in Tamoxifen and Letrozole Treated Breast Cancer Patients

**DOI:** 10.7759/cureus.20030

**Published:** 2021-11-30

**Authors:** Adhari AlZaabi, Hafsa AlAmri, Ghadeer ALAjmi, Manhal Allawati, Fatema Muhanna, Ruqaia Alabri, Fatema AlBusaidi, Shaima AlGhafri, Abdulrahman A Al-Mirza, Khalid Al Baimani

**Affiliations:** 1 Human and Clinical Anatomy, Sultan Qaboos University, Muscat, OMN; 2 Medicine, Sultan Qaboos University, Muscat, OMN; 3 Oncology Department, Sultan Qaboos Comprehensive Cancer Care & Research Centre, Muscat, OMN

**Keywords:** letrozole, aromatase inhibitor, endometrial cancer, tamoxifen, breast cancer

## Abstract

Background: Our study aimed to assess the risk of endometrial pathologies after tamoxifen and aromatase inhibitors (AIs) adjuvant treatment for female breast cancer patients treated at Sultan Qaboos University Hospital in Oman.

Materials and Methods: A total of 457 patients diagnosed with estrogen positive breast cancer between January 2011 and December 2018 were screened. Two hundred and four patients met the inclusion criteria, and their detailed clinicopathological and endometrial surveillance data were collected from their electronic health records.

Results: All patients underwent endometrial assessment during tamoxifen or letrozole therapy. The mean diagnostic age of breast cancer patients is 43.6 years, ranging from 27-84 years. Eighty-three percent of those patients are premenopausal, and 17% are postmenopausal. The mean tamoxifen use duration was 33 months. The majority of patients, 123 (60.3%), have had tamoxifen for three years or less, 47 (23.1%) for 3-5 years, and only 22 (10.8%) were on tamoxifen for more than five years. Increased endometrial thickness was reported in 8% of the premenopausal and 14% of the postmenopausal group. Other endometrial pathologies that were detected are inactive endometrium three (1.47%), atrophic endometrium three (1.47%), serous carcinoma one (0.50%), endometrial cancer two (0.98%), and chronic endometritis one (0.50%), which were not significantly associated with tamoxifen or letrozole therapy duration. Two patients have developed endometrial cancer, and both are postmenopausal and > 60 years old.

Conclusions: Tamoxifen and letrozole did not increase the risk of endometrial cancer in premenopausal patients. Breast Cancer (BC) patients on tamoxifen or letrozole might need a pre-treatment endometrial evaluation and explanation of alarming symptoms to guide further endometrial surveillance.

## Introduction

Since the introduction of tamoxifen as a targeted hormonal therapy for estrogen-receptor-positive (ER+ve) breast cancer in the 1970s, the risk of recurrence and contralateral breast cancer has significantly reduced. Furthermore, the disease-free survival and overall survival rate of ER+ve breast cancer have improved [1-3]. The recommended tamoxifen therapy is up to five years as the annual mortality rate of tamoxifen-treated ER+ve breast cancer patients reduced by 31% after five years of treatment [4]. In 1998, FDA approved tamoxifen to be used as a preventive drug for high-risk women.

Tamoxifen is a selective ER modulator. It has an antagonistic effect on the ER-α receptors in breast tissues and an agonistic effect on the ER- β receptors in the endometrial tissues. This agonistic effect has been shown to increase the rate of several benign and malignant endometrial pathologies such as hyperplasia, atypia, carcinomas, and sarcomas [5-10]. The increased risk of endometrial cancer (EC) is not associated with the daily dose of tamoxifen but with a longer duration and accumulative usage [5,11,12]. The risk has been also found to increase significantly with increasing body weight and postmenopausal status [7,13]. Other studies found no linear relationship between the duration of tamoxifen therapy and its uterotrophic effect [11,12,14]. Therefore, it is not clear yet when to stop tamoxifen and initiate uterine surveillance. Hence, there is an existing debate [15,16] if all breast cancer patients who are receiving tamoxifen should be frequently evaluated for any endometrial changes during the therapy.

On the other hand, aromatase inhibitors (AIs) is another endocrine therapy for breast cancer patients that have reduced breast cancer incidence and improved breast cancer outcome. It has been also found to lower the risk of endometrial cancer compared to tamoxifen [17].

In Oman, about 60% of breast cancer patients are ER+ve [18]. Tamoxifen is usually prescribed to these patients as part of adjuvant treatment for five years or more. AI is given to patients who are postmenopausal and select cases of premenopausal women. In addition, AI is given if tamoxifen is not tolerated or contraindicated. In Sultan Qaboos University Hospital, all tamoxifen and AI-treated ER+ve breast cancer patients undergo frequent endometrial surveillance during the five years of the course of therapy. No prior study has been conducted to evaluate the risk of endometrial pathologies in tamoxifen-treated ER+ve breast cancer patients in Oman. In general, data on tamoxifen use and endometrial cancer risk in the middle east and Asia is scarce. Here we aimed to evaluate such risk in patients treated at Sultan Qaboos University Hospital.

## Materials and methods

Patients

The medical records of 457 ER+ve breast cancer patients who received tamoxifen or aromatase inhibitors at Sultan Qaboos University Hospital between 2011 and 2018 have been retrospectively reviewed. Only 204 patients have met the inclusion criteria, which are patients who are ER+ve and have received tamoxifen and/or AIs, undergone endometrial health assessment for tamoxifen effect evaluation, and have a follow-up visit at the obstetrics and gynecology clinic for endometrial evaluation. Three different AI drugs are used in breast cancer. These include letrozole, anastrozole, and exemestane. In our center, the two commonly used AIs are letrozole and exemestane. Detailed clinicopathological data of all included patients were collected from the electronic health records in a pre-structured datasheet. 

Results and details of different endometrial surveillance tests, pap smear, transvaginal ultrasound, pelvic ultrasound, and endometrial biopsy were collected. Endometrial thickness measured in ultrasound was measured at the site of maximum thickness of the endometrium in a longitudinal plane. The definition of endometrial thickness varies among studies and is different among symptomatic versus asymptomatic and premenopausal versus postmenopausal patients [19]. Here we consider an endometrial thickness of ≥12 mm as thickened endometrium in an asymptomatic premenopausal woman with regular menses [19]. For postmenopausal and amenorrheic premenopausal women, an endometrial thickness ≥5 mm was defined as endometrial thickening. Endometrial biopsy was indicated if the patient has thickened endometrium, abnormal pelvic u/s, or abnormal uterine bleeding.

Additional clinicopathological information relevant to tamoxifen/letrozole therapy was collected, including the dosage and date of commencing and discontinuing treatment. The total duration of tamoxifen/letrozole use was defined as the date of the first prescription to the date of endometrial pathology developed or the last prescription. 

Informed consent was not needed for this study as the data were obtained retrospectively from existing medical records, and no identifying information was collected or used during the analysis. 

Statistical analysis 

Descriptive statistics were used for patients' demographic characteristics analysis. Statistical analysis was performed using the SPSS Inc. Released 2009. PASW Statistics for Windows, Version 18.0. Chicago: SPSS Inc. and Student’s t-test. Age was used to test the association between different parameters, i.e., endometrial findings with age, menopausal status, duration of tamoxifen/letrozole treatment. A P-value <0.05 was considered statistically significant.

## Results

Patient characteristics 

A total of 204 breast cancer (BC) patients, who were on tamoxifen or aromatase inhibitors (AI), are included in this study. Patients’ demographics are listed in Table 1. The mean diagnostic age of breast cancer patients is 43.6 years, ranging from 27-84 years. Eighty-three percent of those patients are premenopausal, and 17% are postmenopausal. The majority (79%) of the patients have received tamoxifen, while 9.3% received AI. 20 mg dose of tamoxifen was given to all the patients in this study, and a 2.5 mg dose of letrozole was given. During data collection, the majority of patients, 123 (60.3%), have had tamoxifen for three years or less, 47 (23.1%) for 3-5 years, and only 22 (10.8%) were on tamoxifen for more than five years. The mean tamoxifen use duration was 33 months. 8.8% of the BC patients were grade 1, 58.8% were grade 2, and 25.5% were grade 3. 

**Table 1:** Demographics of breast cancer patients on tamoxifen or letrazole

Category	Number of patients	
Total number of included patients	204	
Age (years)	Mean: 43.6 Range: 27-84	
Menopausal status	Premenopausal: 169 (82.8 %) Postmenopausal: 35 (17.2%)	
Duration of tamoxifen and letrazole therapy (months)	Tamoxifen therapy	Letrazole therapy
Less than three years	123 (60.3)	16 (7.8)
3-5 years	47 (23.1)	7 (3.4%)
More than five years	22 (10.8)	2 (0.1)
Grade of breast cancer	Grade1: 18 (8.8%) Grade2: 120 (58.8%) Grade3: 52 (25.5%) NA: 17 (6.9%)	
HER2 status	HER2 positive: 74 (36.2%) HER2 negative: 127 (62.2)	
PR (progesterone receptors) status	PR positive: 174 (85.3%) PR negative: 24 (11.7%) NA: 6 (3.0%)	
Breast cancer histology	Ductal: 168 (82.3%) Lobular: 22(10.8%) Adenocarcinoma: 1 (0.5%)	

62.2% were human epidermal growth factor receptor-2 (HER2) negative, and 85.3% of patients are were progesterone receptors (PR) positive. The majority of patients (82.3%) have had ductal BC followed by lobular BC (10.8%). Body mass index information was not available for the majority of patients. 

**Figure 1:**
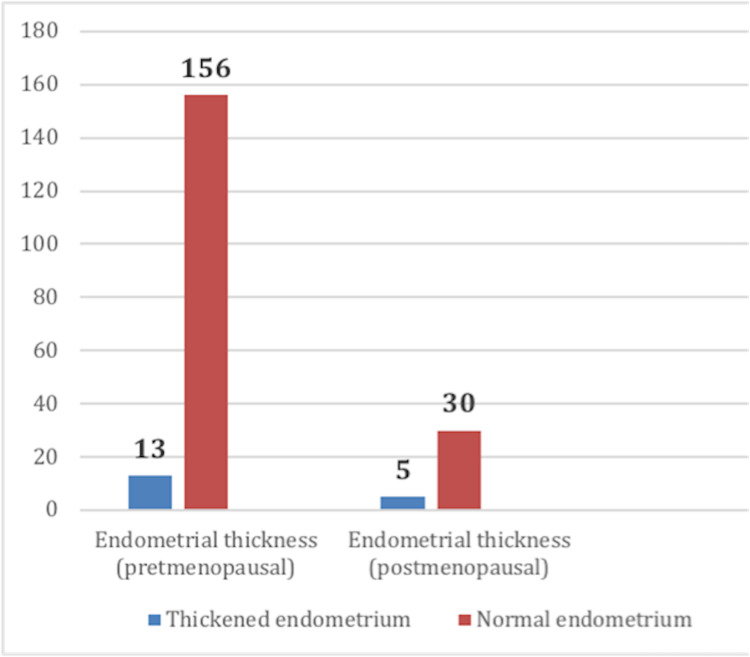
Pelvic/transvaginal ultrasound results. * Endometrial thickness ≥12 mm was defined as thickened endometrium for premenopausal women, while endometrial thickness ≥5 mm in postmenopausal.

Patients have routine endometrial assessments at different intervals. The time of the first gynecological assessment since starting tamoxifen varies greatly among patients with a median period of 13 months. One hundred and ninety-two (94.1%) of the included patients had a pap smear as part of the endometrial assessment, 157 (76.9%) of the patients have had negative results, while nine (4.4%) had atypia (Figure 1). 

**Figure 2:**
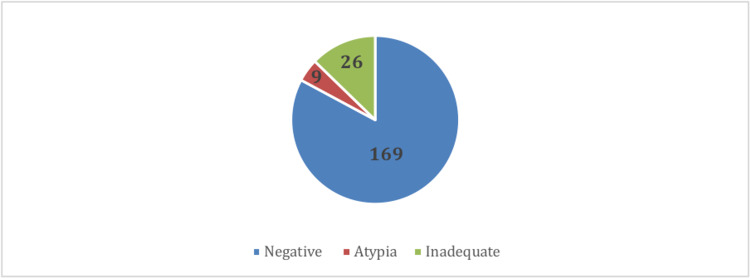
Pap smear results of all patients

**Figure 3:**
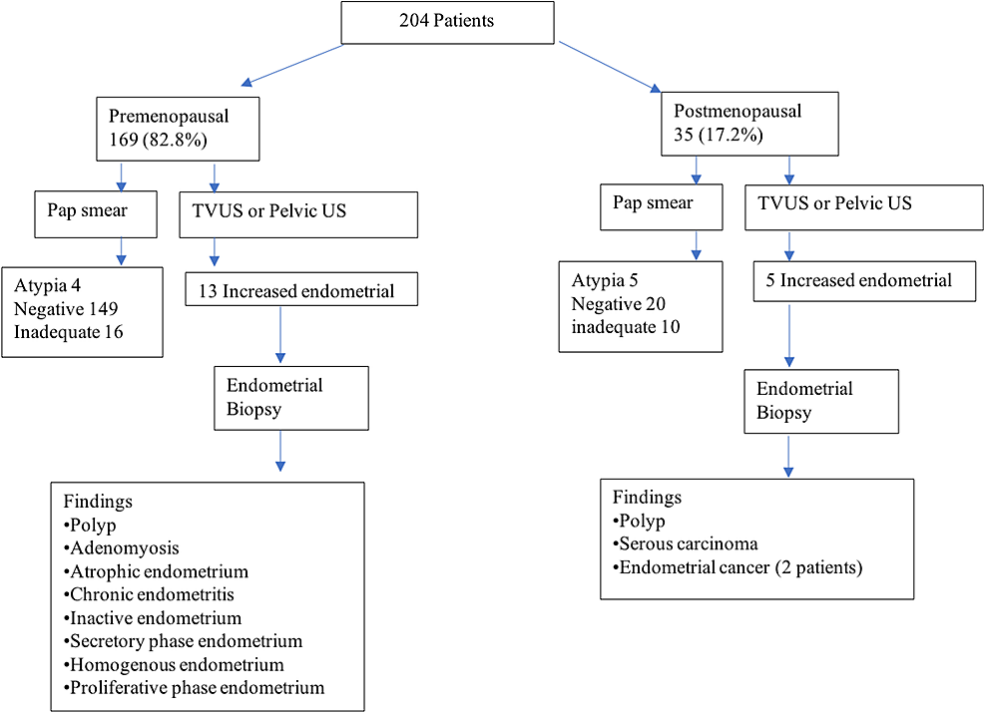
Summary of the study's findings

All patients have had ultrasound evaluation (either pelvic US or transvaginal (TV)-US), and 10.3% underwent uterine biopsy. The prevalence of endometrial thickening was 8% in the premenopausal and 14% in the postmenopausal group (Figure 2). Patients with thickened endometria underwent hysteroscopy and biopsy. Endometrial pathologies that were detected are three (1.47%) inactive endometrium, three (1.47%) atrophic endometrium, one (0.50%) serous carcinoma, two (0.98%) endometrial cancer, and one (0.50%) chronic endometritis, which were not significantly associated with tamoxifen or letrozole therapy duration, age or menopausal status (p-value >0.05). The two patients (0.98%) who were found to have endometroid cancer are both postmenopausal and above 60 years. One of them was on tamoxifen for 26 months at diagnosis, and the other one was on letrozole for about 28 months at diagnosis. Figure 3 summarizes the findings of the study. The t-test showed no significant association between increased endometrial thickness with age, duration of tamoxifen use, or breast cancer hormonal status regardless of the menopausal status (P-value 0.2, 0.09, 0.1, respectively).

## Discussion

Despite the growing evidence of tamoxifen-related increased risk of endometrial cancer, it remains the standard adjuvant hormone treatment for women with estrogen receptor (ERα+) breast cancer. This is because the net benefit of tamoxifen greatly outweighs the risk. The risk of endometrial cancer in tamoxifen is highly associated with postmenopausal women, body mass index, the existence of pre-treatment endometrial lesions, and prolonged use [19,20]. In this study, most patients are premenopausal, and they all have endometrial surveillance either a pap smear and transvaginal U/S or the pelvic US after starting the adjuvant therapy. Patients found to have thickened endometrium were further evaluated by hysteroscopy and biopsy. Evaluation of endometrial thickness in asymptomatic patients did not offer an early diagnosis of any endometrial pathology. Theoretically, tamoxifen does increase endometrial thickness due to the sub-endometrial gland hypertrophy without any atypical transformation [20-23]. Therefore, tamoxifen-induced endometrium thickness does not necessitate further unnecessary, costly, and potentially harmfully invasive procedures [24,25]. Gerber et al. reported identifying one uterine cancer in hysteroscopies done for 52 asymptomatic women found to have thickened endometrium (> 9mm) on TV U/S [21]. They reported four uterine perforations as a result of the performed hysteroscopies. 

Aromatase inhibitors have been found to reduce the risk of endometrial cancer [17,22]. Furthermore, it abrogates the risk exerted by tamoxifen once it is replaced by the AIs [17,22]. 

In this study, very few patients have developed benign endometrial pathologies that were not significantly associated with menopausal status, age, duration of treatment, or HER2 status (p-value >0.05). Two patients have developed endometrial cancer, and both were postmenopausal and > 60 years old. As per the records, there is no pretreatment endometrial assessment done for these patients to check any pre-existing endometrial pathology. Berlière and co-workers [21,23] reported an association between baseline pre-treatment endometrial pathologies and endometrial hyperplasia and atypia. 

These findings are consistent with findings from other studies that reported a low risk of EC in women <50 years [24]. Available evidence has divided ER+ve breast cancer patients into low-risk and high-risk groups. High-risk women are those who are postmenopausal and have an endometrial abnormality at baseline [21,23]. Premenopausal women appear to represent a low-risk group unless symptomatic [11,13,25,26]. Therefore, routine endometrial assessment with endometrial transvaginal ultrasonography and biopsies for the detection of uterine cancer in asymptomatic premenopausal and postmenopausal women may not be cost-effective and might lead to unnecessary invasive procedures [27]. This has been translated into the guidelines published by both the American [28] and Royal Australian and New Zealand Colleges of Obstetricians and Gynaecologists, which do not recommend routine surveillance in asymptomatic patients receiving tamoxifen.

Instead, a baseline the pre-treatment hysteroscopic assessment of ER+ve breast cancer patients would be valuable to plan if further endometrial assessments are needed. Supporting this concern is the finding by Garuti et al. that 50% of patients who had hyperplasia without atypia prior to tamoxifen have progressed to an advanced stage lesion after 24 months of tamoxifen use [29]. 

Limitation

This study is a single institute study that doesn’t represent the whole population. The sample size is relatively small, which might impact the results. Most patients have a follow-up period of up to 3-5 years, while endometrial changes might happen after the completion of the five years plan. The cut-off for endometrial thickness differs among studies; therefore, comparison between studies was challenging.

## Conclusions

In conclusion, a detailed counselling and uterine assessment for breast cancer patients before starting tamoxifen or letrozole treatment are needed to explain the risk and all alarming gynecological symptoms. All patients who are on tamoxifen or letrozole should be properly investigated once they experience abnormal gynecological symptoms.
